# Open(ing) Access: Top Health Publication Availability to Researchers in Low- and Middle-Income Countries

**DOI:** 10.5334/aogh.3904

**Published:** 2023-06-05

**Authors:** John L. Kilgallon, Saumya Khanna, Tanujit Dey, Timothy R. Smith, Kavitha Ranganathan

**Affiliations:** 1 Computational Neurosciences Outcomes Center, Department of Neurosurgery, Brigham and Women’s Hospital, Harvard Medical School, Boston, MA, United States; 2 Brigham and Women’s Hospital, Harvard Medical School, Boston, MA, United States; 3 Center for Surgery and Public Health, Brigham and Women’s Hospital, Harvard Medical School, Boston, MA, United States; 4 Division of Plastic and Reconstructive Surgery, Brigham and Women’s Hospital, Harvard Medical School, Boston, MA, United States

**Keywords:** LMICs, Open Access, Surgery, Publishing, Global Health

## Abstract

**Introduction:**

Improving access to information for health professionals and researchers in low- and middle-income countries (LMICs) is under-prioritized. This study examines publication policies that affect authors and readers from LMICs.

**Methods:**

We used the SHERPA RoMEO database and publicly available publishing protocols to evaluate open access (OA) policies, article processing charges (APCs), subscription costs, and availability of health literature relevant to authors and readers in LMICs. Categorical variables were summarized using frequencies with percentages. Continuous variables were reported with median and interquartile range (IQR). Hypothesis testing procedures were performed using Wilcoxon rank sum tests, Wilcoxon rank sum exact tests, and Kruskal-Wallis test.

**Results:**

A total of 55 journals were included; 6 (11%) were Gold OA (access to readers and large charge for authors), 2 (3.6%) were subscription (charge for readers and small/no charge for authors), 4 (7.3%) were delayed OA (reader access with no charge after embargo), and 43 (78%) were hybrid (author’s choice). There was no significant difference between median APC for life sciences, medical, and surgical journals ($4,850 [$3,500–$8,900] vs. $4,592 [$3,500–$5,000] vs. $3,550 [$3,200–$3,860]; p = 0.054). The median US individual subscription costs (USD/Year) were significantly different for life sciences, medical, and surgical journals ($259 [$209–$282] vs. $365 [$212–$744] vs. $455 [$365–$573]; p = 0.038), and similar for international readers. A total of seventeen journals (42%) had a subscription price that was higher for international readers than for US readers.

**Conclusions:**

Most journals offer hybrid access services. Authors may be forced to choose between high cost with greater reach through OA and low cost with less reach publishing under the subscription model under current policies. International readers face higher costs. Such hindrances may be mitigated by a greater awareness and liberal utilization of OA policies.

## Introduction

Equitable access to information is vital to global health. Over one million scientific articles are pirated each year. Among these, 69% of download requests come from low- and middle-income countries (LMICs) [1]. Incomplete access to current literature in LMICs precludes the delivery of high-quality care for diverse patient populations, impedes scientific progress, and encourages alternative, back door methods of acquiring educational material [2]. While many clinical trials are performed on patients from LMICs, findings from these studies are not consistently accessible to these same populations [3,4,5]. Unfortunately, LMIC researchers and research institutions face numerous barriers from the standpoint of authorship and readership [6]. For example, between 2012 and 2016, only one third of the global top 500 cited emergency medicine articles were freely accessible [7,8].

Illegal online research libraries such as Sci-Hub have emerged to circumvent insufficient access to scientific literature [1]. Data on pirated scientific literature represents an opportunity for publishers interested in broadening their impact and profit; increasing access to current literature at a lower cost for authors from LMICs may augment revenue currently lost to piracy. Open Access (OA) policies will allow for greater citations and therefore greater journal exposure, as more people can read the research. In addition to the monetary benefits, increased access to research and knowledge can foster greater scientific education and literacy globally, having positive spillover effects into public policy [9].

Legal forms of access to scientific literature include the World Health Organization’s Health InterNetwork Access to Research Initiative (HINARI) and OA publishing. While HINARI facilitates access to current literature among those from the lowest income countries, many scientists and clinicians from LMICs fall above this threshold and are therefore ineligible [1]. For example, Sci-Hub is also highly utilized by upper-middle income nations including India, China, Brazil, and Iran [1,10]. Reasons for pirating in these countries include insufficient funding for continuing education, policy or government restrictions, and lack of awareness around expected publication and subscription practices [1]. Additionally, this program does not account for socioeconomic status at an individual level, and is instead focused on country-level income categorization. In fact, authors from LMICs require a larger proportion of their available income to publish OA [5]. Encouraging authors to publish in OA formats is another solution to the problem of insufficient access to current scientific literature. Open access publishing provides immediate availability of literature in an equitable manner that benefits all stakeholders. Unfortunately, many institutions do not cover the cost of publishing in OA formats; authors must use personal funds or grant support to publish in a manner that guarantees OA [11].

There are barriers that limit the ability of authors and readers in LMICs to access current scientific literature. As OA is one potential approach to improving access to scientific literature in LMICs, the goal of the current study is to define OA policies, subscription costs, and availability of health literature as relevant to people in LMICs. First, we assessed top journals’ policies surrounding OA. We focused on these journals specifically as we believe that it is most important for the highest levels of evidence to reach those in LMICs. Next, we evaluated how these policies differ for surgical and non-surgical journals to characterize differences in access based on clinical subject matter. Finally, we separated non-surgical journals further into medical sciences and life sciences to uncover any differences between the three groups. By evaluating current policies through the lens of those in low resource environments, it may be possible to optimize practices in a manner that empowers publishers, authors, and readers to act collaboratively to increase access to relevant and potentially life-saving information.

## Methods

### Journal selection

We performed a cross-sectional analysis from data collected on OA policies from scientific journals. Top journals were identified using the Google Scholar Top Publication database, an online catalogue of the most impactful academic journals as determined by h-5 index, which is the largest number “h” such that “h” articles published by the journal in the last five years have at least “h” citations each. We included only the highest reach publications because they are the journals that are most likely to affect clinical practice and scientific guidelines, and thus are likely to be most meaningful for LMIC authors and readers. We selected the 20 top journals in the “Life Sciences & Earth Sciences” category (e.g., *Nature*), the 20 top journals in the “Health & Medical Sciences” category (e.g., *The New England Journal of Medicine*), the 20 top journals in the “Surgery” subcategory (e.g., *JAMA Surgery*).

### Relevant variables

Variables relevant to LMIC authors included each Journal Impact Factor (IF), calculated by Clarivate [12], and publicly available OA policies. Variables relevant to LMIC readers included subscription costs, current level of free to access articles in the most recent issue of the journal, and availability on HINARI, a database of publishers willing to grant LMICs free access to select journals and articles. From the authors’ perspective, the IF indicates the potential reach of their research, and the APCs required by some journals to publish with OA were used as a metric to evaluate costs incurred to disseminate research. From the readers’ perspective, subscription prices demonstrate affordability to the journals researched in our study; availability on HINARI demonstrates accessibility specifically for LMIC readers.

### Open access policies

To define OA policies, we used the SHERPA RoMEO database, an online resource that aggregates and analyzes publisher OA policies and each journal’s publicly available publishing protocols. Policies where a payment is required by either the author or reader include the Gold OA model, subscription model, and hybrid model; Green OA and HINARI do not require payment by either party. Gold OA journals publish articles with immediate access after payment of APCs by the authors. Subscription journals publish articles free of significant charge to authors and provide access only to readers who pay subscription or pay-per-view fees, which range from journal to journal. Hybrid journals are those that allow authors the option to publish either by the subscription model, with little to no publishing fees, or with an APC, making the article Gold OA upon its publication. Delayed OA offers full access after an embargo period usually of 6–12 months. Subscription fees and APCs were recorded in US dollars. The Green OA model involves the use of platforms such as arXiv.org, an open-access repository of electronic preprints and post-prints approved for posting after moderation (but not peer review), to publish the accepted or submitted versions of a manuscript free of charge. The use of the paid models and Green OA is not mutually exclusive. Availability on HINARI was assessed by determining whether each journal’s publishing company was listed on Research4Life’s website as a “HINARI Partner.” Finally, in order to gauge each journal’s current level of accessibility to those in LMICs, the most recent issue of each journal was evaluated for the percentage of articles freely available.

### Statistical analysis

Categorical variables were summarized using frequency with percentage. Continuous variables were reported as median and interquartile range (IQR). Nonparametric tests like Wilcoxon rank sum test, Wilcoxon rank sum exact test (for two sample comparisons), Kruskal-Wallis test (ANOVA or more than two-samples comparisons) were performed in case of hypothesis testing status quo. Significance of all hypothesis tests was considered at alpha level of significance less than 5%. All analyses were performed with R software version 4.1.0 (https://cran.r-project.org/).

## Results

### Open access policies

**Figure 1:**
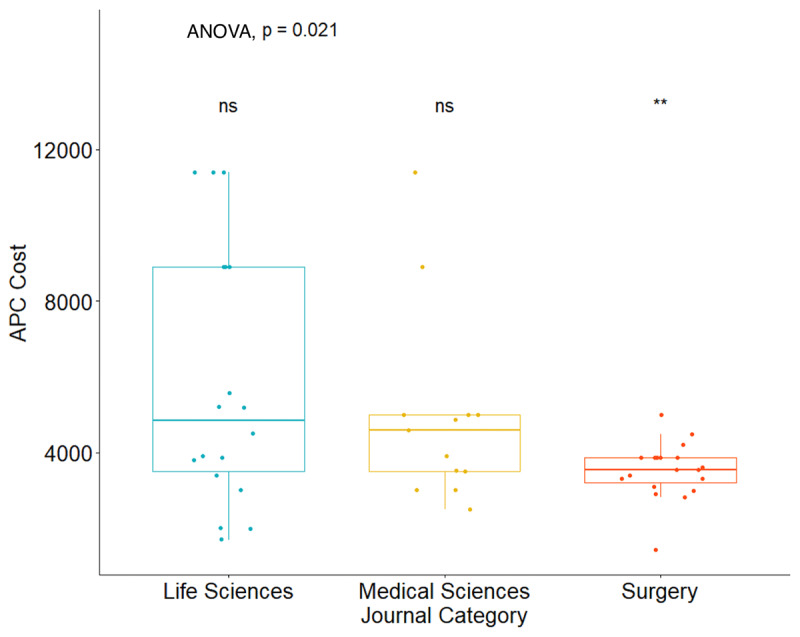
Comparison of APC costs across journal type. Articles without APC options are excluded from this figure.

A total of 55 journals were included after the removal of duplicates due to category overlap. Of these, 6 journals (11%) were classified as Gold OA, 2 (3.6%) were defined as subscription only journals, 4 (7.3%) were delayed OA, and 43 (78%) were defined as hybrid (Table 1, Figure 1). A total of 50 journals (91%) offered an option to self-publish with Green OA (no payment required by authors or readers), and 47 (85%) were considered to be HINARI partners. We found that that the median percentage of articles in surgical, life sciences, and medical journals that were freely available to readers was 14%, although this result was not statistically significant.

**Table 1:** Summary statistics of all the characteristics of the data collected for the analysis.


CHARACTERISTIC	N = 55^1^

Journal Impact Factor	12 (4, 26)

h5 Index	138 (65, 174)

Life Sciences	20 (36%)

Medical	15 (27%)

Surgical	20 (36%)

HINARI Partner	47 (85%)

Gold OA Journal with APC	6 (11%)

Green OA Journal	50 (91%)

Delayed OA Journal	4 (7.3%)

Hybrid Journal	43 (78%)

Subscription Only Journal	2 (3.6%)

OA Fees APC	50 (93%)

APC (USD)	3,860 (3,300, 5,000)

Individual Subscription Price – United States (USD)	338 (213, 518)

Individual Subscription Price – International (USD)	370 (216, 584)

Institutional Subscription Price – United States (USD)	1,931 (1,530, 2,934)

Higher International Subscription Price (USD)	17 (42%)


^1^ Median (IQR); n (%).

### Relevance to authors

**Figure 2:**
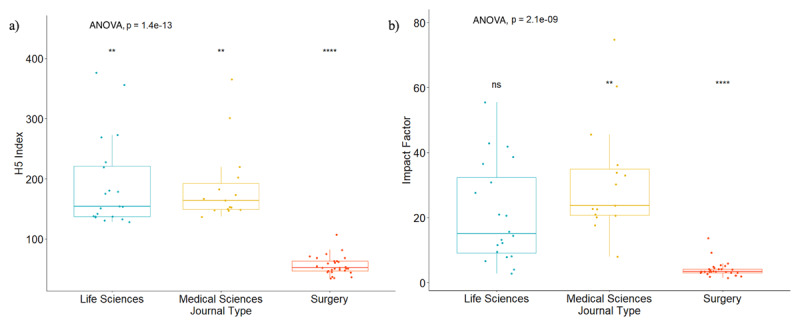
Comparisons across journal type for: **a)** h-5 index; and **b)** Journal Impact Factor.

**Figure 3:**
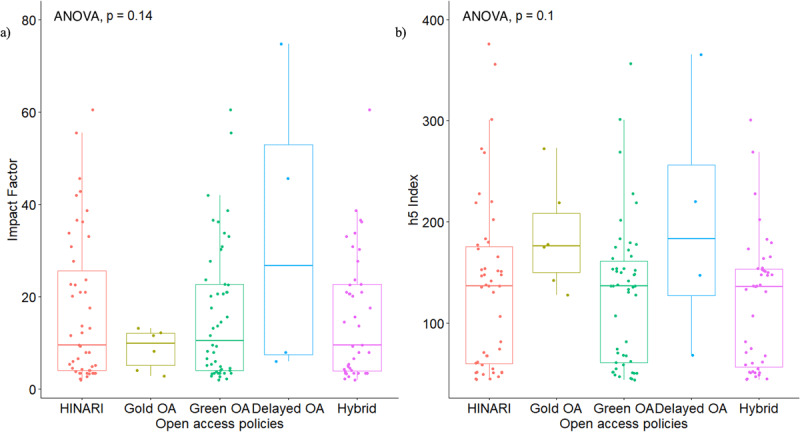
Comparisons across journal Open Access policies for: **a)** h-5 index; and **b)** Journal Impact Factor.

The median APC was the highest for life sciences journals at $4,850 ($3,500–$8,900), followed by medical journals at $4,592 ($3,500–$5,000), and surgical journals at $3,550 ($3,200–$3,860) (p = 0.054) (Table 2, Figure 1). The median h-5 index was 154 (137–221) for life sciences journals, 164 (149–192) for medical sciences journals, and 54 (50–68) for surgical journals (p < 0.001) (Table 2, Figure 2). The median h-5 index was 2.85 and 3.04 times higher for life sciences and medical journals compared to surgical journals, respectively, meaning that the largest number “h” such that “h” articles published in the last five years have at least “h” citations is about three times greater in life sciences and medical journals than in surgical journals. The median IF was 15 (9–32) for life sciences journals, 24 (21–35) for medical sciences journals, and 4 (3–5) for surgical journals (p < 0.001) (Table 2, Figure 2). OA policies did not seem to affect journal reach, as there was no significant difference across journal OA policies in relation to IF (p = 0.140) or h-5 index (p = 0.100) (Figure 3).

**Table 2:** Individual assessments of several characteristics with respect to three different subject domains.


CHARACTERISTIC	LIFE SCIENCES, N = 20^1^	MEDICAL SCIENCES, N = 15^1^	SURGERY, N = 20^1^	P-VALUE^2^

Journal Impact Factor	15 (9, 32)	24 (21, 35)	4 (3, 5)	<0.001

h5-Index	154 (137, 221)	164 (149, 192)	54 (50, 68)	<0.001

APCs (USD)	4,850 (3,500, 8,900)	4,592 (3,500, 5,000)	3,550 (3,200, 3,860)	0.054

Individual Subscription Price – USA (USD)	259 (209, 282)	365 (212, 744)	455 (365, 573)	0.038

Individual Subscription Price –International (USD)	259 (209, 282)	378 (216, 962)	514 (374, 618)	0.052

Institutional Subscription Price – USA (USD)	9,850 (6,933, 12,768)	2,338 (1,938, 4,326)	1,587 (1,324, 2,052)	0.025


^1^ Median (IQR). ^2^ Kruskal-Wallis rank sum test.

### Relevance to readers

The median US individual subscription cost (USD/Year) was $259 ($209–$282) for life sciences journals, $365 ($212–$744) for medical journals, and $455 ($365–$573) for surgical journals (p = 0.038) (Table 2). The international individual subscription costs were $259 ($209–$282) for life sciences journals, $378 ($216–$962) for medical journals, and $514 ($374–$618) for surgical journals (p = 0.052). The cost of an institutional subscription, which may be purchased for use by large groups such as hospitals or universities, was $9,850 ($6,933–$12,768) for life sciences journals, $2,338 ($1,938–$4,326) for medical journals, and $1,587 ($1,324–$2,052) for surgical journals (p = 0.025). Finally, 17 journals (42%) had a subscription price that was higher for international readers than for US readers (Table 1).

## Discussion

Our results show that there is inequality in costs to access publications for US and international readers. International readers pay more on average to subscribe to surgical journals than their US peers, which is especially burdensome for those in LMICs due to insufficient funding for healthcare personnel and scientists’ continuing education. Article processing charges for authors, in general, are higher for non-surgical journals than for surgical journals. Although the h-5 index and Journal Impact Factors are also higher on average for non-surgical journals, authors are nevertheless required to pay an exorbitant price for publication with Open Access. The burden continues to be disproportionate for those in LMICs because authors from LMICs require a larger proportion of their available income to publish OA and, historically, funding from institutions or universities covering APCs in LMICs is low [5,13].

### Authors

Current policies are heavily skewed towards a hybrid model; authors seeking to publish in top journals may be forced to choose between high cost with greater reach publishing under the Gold OA model and low cost with less reach publishing under the subscription model. This can be an issue for many LMIC authors, because although fully Gold OA Journals will waive APCs for LMIC authors, hybrid journals do not have uniform policies on this issue. Additionally, it has been shown that while a majority (72.9%) of publications from low-income countries are published with OA, authors from high-income countries utilize these options only 45.1% of the time [14]. This indicates that LMIC authors understand the importance of publishing with OA. However, as the journals in this study require such high APCs, LMIC authors can lose incentives to publish regularly in top-tier journals with OA, causing an imbalance in the flow of information in research.

Our results show that the median APC is highest for life sciences journals and lowest for surgical journals. However, publishing costs bear little correlation with the impact of the journal; paying a larger APC does not necessarily correlate to a greater number of citations [15]. Moreover, OA articles on average receive 18% more citations than non-OA articles [16]. Currently, however, a majority (69.0%) of global health research publications are not freely available online, and 60.8% of researchers do not self-archive their work with Green OA [13]. Implementing OA policies can help shift incentives. Expanding “Waive Fees” policies at hybrid journals for LMIC authors and advocating for greater utilization of Green OA through repositories such as arXiv.org are alternative solutions. Additionally, annual research budgets from governments or international bilateral/multilateral donors can include a line item to cover publishing fees for those from LMICs [17].

### Readers

LMIC readers of top journals are forced to choose between exorbitant subscription costs and HINARI, a resource which is promising for the future if deployed correctly, but one which is currently underutilized. Our results showed that surgical journals are more likely to have a higher international subscription price compared to non-surgical journals. Professionals and researchers in LMICs interested in learning about surgery face skewed barriers in accessing published literature, putting them at a great disadvantage. This is concerning, as research shows that life-saving surgical and anesthesia care in low-income and middle-income countries (LMICs) has stagnated or regressed [18]. The development and delivery of surgical care in LMICs is under-prioritized in global health [18]. Health professionals should be vigilant towards the fact that LMICs, which have a great need to access more surgical information, have a skewed disadvantage in accessing published research. Strategies to ensure equitable access must expand beyond sole reliance on HINARI given variable awareness of this resource among those in LMICs.

In a study of 1,150 clinicians and researchers in 12 tertiary health institutions that had access to HINARI, only 35.1% had formal training on how to use it, and 50.0% encountered problems in accessing resources [19]. Moreover, poor internet connectivity and poor electricity have been cited as barriers to using HINARI [20]. In addition to facing challenges in a campus setting, LMICs may face unique challenges in their country at-large, including lack of funding, poorly developed national research programs, and a lack of cohesion between research and policy-making. Research also shows that some LMICs are emerging from decades of conflict and have lost skills and experience to other countries or regions through displacement or economic necessity [21]. Finally, the presence of variation in waiver guarantees may be particularly challenging for non-English speakers who might be at a greater disadvantage when navigating and requesting these waivers from English journals [22]. Variability in policies may also create a backlog for editorial boards that oftentimes receive many requests. Possible future directions include increasing LMIC readers’ awareness of access options by creating tools to help people find OA versions published through Green OA and improving the HINARI interface.

### Limitations

There are several limitations to this study. Importantly, some journals do present policies by which they are willing to accept requests for waive-fees for those in need. However, we were unable to include these policies in our analysis as there is lack of standardized guidelines and public awareness of these options. Other limitations include the fact that only top journals that are published in English were evaluated. Additionally, subscription costs and APCs are subject to change. The Health InterNetwork Access to Research Initiative is used as the primary method of evaluating greater access to publications for LMICs; however, not all institutions in LMICs use HINARI, and there may be other programs available of which we are unaware. Finally, lower-profile journals were excluded in the analysis, meaning that these trends may not be the same across all levels of journals.

## Conclusions

There are currently cost-related barriers for scientific authors and readers in LMICs. This leads to a skew in the dissemination of information, with growth and progress in the fields of global health and global surgery likely suffering in two dimensions. The first is a lack of diversity in authorship. The second is LMIC readers facing limited access to information due to barriers in accessing published scientific literature. This is especially concerning, given that scientific knowledge and access to current guidelines is essential for global health [18]. This can be mitigated by a greater awareness and more liberal utilization of OA. The implications of equity in research have a profound impact, and can potentially catalyze new innovations accessible to a diverse group of stakeholders.
